# Root Cultures, a Boon for the Production of Valuable Compounds: A Comparative Review

**DOI:** 10.3390/plants11030439

**Published:** 2022-02-05

**Authors:** Masooma Jawad Hussain, Yawar Abbas, Naushaba Nazli, Sara Fatima, Samantha Drouet, Christophe Hano, Bilal Haider Abbasi

**Affiliations:** 1 Department of Biotechnology, Quaid-i-Azam University, Islamabad 45320, Pakistan; masoomajawad@bs.qau.edu.pk (M.J.H.); ayawar@bs.qau.edu.pk (Y.A.); naushabanazli@bs.qau.edu.pk (N.N.); sarafatima@bs.qau.edu.pk (S.F.); 2 Laboratoire de Biologie des Ligneux et des Grandes Cultures (LBLGC), University of Orleans, INRAE USC1328, F28000 Chartres, France; samantha.drouet@univ-orleans.fr (S.D.); hano@univ-orleans.fr (C.H.)

**Keywords:** adventitious root culture, hairy root culture, medicinal plants, elicitation strategy, bioreactor

## Abstract

Medicinal plants are an inevitable source of pharmaceutical drugs and most of the world population depends on these plants for health benefits. The increasing global demand for bioactive compounds from medicinal plants has posed a great threat to their existence due to overexploitation. Adventitious root and hairy root culture systems are an alternative approach to the conventional method for mass production of valuable compounds from medicinal plants owing to their rapid growth, biosynthetic and genetic stability. The main purpose of this review is to investigate the recent scientific research published worldwide on the application of adventitious and hairy root cultures to produce valuable compounds from medicinal plants. Furthermore, a comparison of adventitious root vs. hairy root cultures to produce valuable compounds has also been discussed. Various aspects such as medium composition, carbon source, pH, amount of macronutrients, optimization strategy, scale-up cultures, and use of biotic abiotic and nano-elicitors at various concentrations are the topic of discussion in this review. Several studies on adventitious and hairy root cultures of *Polygonum multiflorum*¸ *Withania somnifera*¸ *Echinacea purpurea* and *Ajuga bracteosa* have been discussed in detail which highlights the importance of elicitation strategies and bioreactor system, presenting commercial applications.

## 1. Introduction

Medicinal plants are an inevitable source of pharmaceutical drugs and most of the world population depends on these plants for their health. Plant cell and tissue cultivation to produce pharmaceutically important components of commercial interest have gained popularity over the last few years [1]. To save lives, humans have used medicinal plants as an endless source of drugs. In recent years plant cells, tissues or organs have increasingly been cultivated to get compounds that are both pharmaceutically and economically valuable [2]. After being isolated by solvent extraction from plants (that are grown normally) these compounds are then used as pharmaceuticals, nutraceuticals, pigments, foodstuffs, and cosmetics. Increasing requirements of secondary metabolites have diverted the attention towards cell culture technology to bring possible changes in secondary metabolism to produce bioactive molecules [3]. However, secondary metabolite production is suitable neither by cell culture nor from naturally grown plants. Major hurdles are higher content of water in cells, foaming, wall growth, and unstable production of metabolites in bioreactors [4]. In comparison, metabolite biosynthesis in naturally grown plants is affected by species or genus or might be activated just during a specific growth and developmental stage or by the availability of nutrients and pesticide contamination furthermore a great danger is imposed on the existence of plant species as these are continuously been destroyed for medicines [5]. Now, it is clear that for great socio-economic value other complementary techniques for complete plant cultivation should be developed to produce biologically valuable secondary metabolites. These are the reasons why efforts have been made in past years to develop plant cell, tissue, and organ culture as an alternative method to plant cultivation to produce secondary metabolites of elite pharmacological values [3].

Recent plant research advancements depict that to produce biologically active compounds, the cultivation of adventitious roots in a bioreactor is a great alternative to other methods. A greater rate of proliferation, enormous potentialities of accumulation, and stable production of important secondary metabolites result when adventitious roots are induced in phytohormone supplemented medium under sterile conditions [6]. Considering these issues for mass scale cultivation of adventitious roots by employing bioreactor technology is utilized to produce important plant-derived secondary metabolites. Monitoring of parameters such as pH temperature and the oxygen concentration in the bioreactor can be done; therefore, bioreactor culture system is better than a traditional tissue culture system [7]. The nutrient concentration can be adjusted, and nutrient uptake can also be increased by continuous medium circulation. Moreover, cost and time can be decreased by improving cell proliferation and regeneration rates, product quality can also be controlled, the product 3 can be free of pesticide contamination, and the product can be obtained all year round to meet the increasing global requirement [8]. Despite the great advantages of secondary metabolite production by plant cell cultures, only paclitaxel, shikonin, ginsenosides, and berberine have been produced on a commercial scale, and those process plants are located in the USA, Japan, South Korea, and China, respectively [9]. To produce immense quantities of the plant-derived bioactive molecules for use in a variety of human therapy and cosmetics, adventitious root culture using automated bioreactor technology of various medicinally important plants must be established.

In the present study, the establishment of adventitious and hairy root cultures for the production of valuable compounds, and the recent progress in secondary metabolite production, from medicinal plants, by employing various enhancement strategies have been discussed. Furthermore, highlights regarding the comparison of adventitious vs. hairy roots for the production of valuable compounds have also been mentioned.

## 2. Adventitious Root Culture

Roots are regarded as a part of great secondary metabolism in the whole plant because these are a good source for bioactive molecules, proteins, and a range of metabolites [10]. Recent advances in plant biotechnology enable us to culture plant cells, tissues, and organs instead of complete plants to produce valuable secondary metabolites [11]. Because the low productivity and instability of plant cultures and non-synthesis of few important compounds in undifferentiated cells, mass production of important bioactive molecules is considered commercially difficult by plant cell culture [12]. Keeping these issues in consideration, to produce pharmaceutical and nutraceutical secondary metabolites, the adventitious root culture in a large-scale bioreactor is regarded as a great approach.

In an appropriate phytohormone supplemented medium and aseptic conditions, adventitious roots displayed a greater growth rate and a more active secondary metabolism [13]. Additionally, these are suitable biological materials for good commercial production of great secondary metabolites without foreign genes under in vitro conditions. Adventitious roots displayed greater stability in their growing environment and produced a cosmic amount of secondary metabolites in intracellular spaces and it can be more easily extracted and can be cultivated in a phytohormone amended medium with less inoculum but a greater growth rate compared to cell cultures [14].

One of the major steps of in vitro propagation is adventitious root formation which is used for the cultivation of several plant crops, including medicinal plants. As a result of new advancements in propagation technology, large amounts of biologically important compounds, such as phenols, terpenoids, and alkaloids have been produced using plant cells, tissue, as well as organ cultures [15]. Adventitious root cultures are thought to be the most promising method for biomass production because of their increased growth rate and stable metabolites production [16]. For example, adventitious roots of many important medicinal plants cultivated by micropropagation showed increased biomass production, accumulation, and biologically active compounds production. Therefore, in vitro culture of adventitious roots has enormous potential to be developed on a large scale for the production of biologically active compounds. Furthermore, plant roots are the main raw materials used for herbal drug preparations, accounting for about 60% of herbal medicinal plants used in ethnomedicine. Because of its capability for micropropagation and germplasm preservation, an adventitious root culture is highly useful [17].

## 3. Hairy Root Culture

Hairy root cultures hold immense potential for the biosynthesis of various classes of influential secondary metabolites along with volatile organic compounds. The production of fine roots occurs when *Agrobacterium rhizogenes* inserts its T-DNA into the Ri plasmid to genetically transform the plant tissue. The main advantage of the hairy root culture method is that plant growth regulators do not require media supplementation because the inserted T-DNA contains the required genes for endogenous auxin production. Hairy roots are highly branched as they lack geotropism and can, therefore, grow faster than normal roots. In addition to the typical root metabolites, they also produce metabolites that are naturally secreted by aerial parts of the plants. Moreover, like any other cell culture technology, hairy roots are physiologically and biochemically stable. They display faster growth, spontaneous shoots generation, as well as chemical and morphological similarity to the roots of a wild-type plant. These properties make hairy root cultures extremely convenient for research and industry [18,19,20,21].

## 4. Hairy Root vs. Adventitious Root Culture

Nowadays, studies on the production of crucial bioactive compounds in hairy root culture are increasingly popular. *Agrobacterium rhizogenes* is an agent which is responsible to cause hairy roots in plants characterized by a higher growth rate and genetic stability [22]. Despite the high growth rate, biochemical and genetic stability exhibited by hairy roots, the secondary metabolites produced in hairy root liquid culture remain in the root tissues which affect the cell growth and pose difficulties in extraction during the downstream process. While adventitious root cultures release secondary metabolites into the culture medium which can be easily extracted [23].

**Figure 1:**
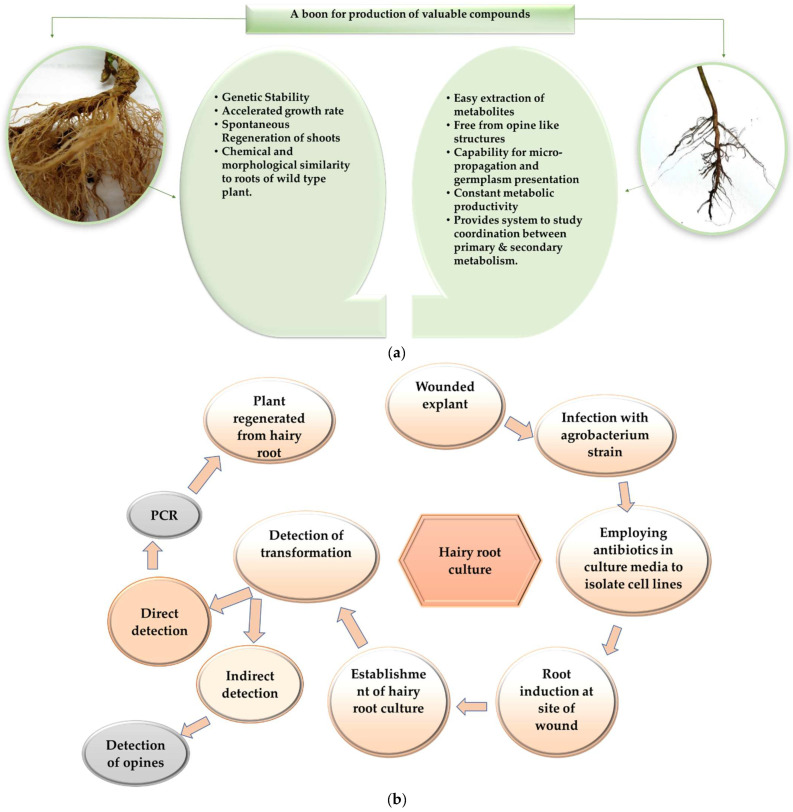

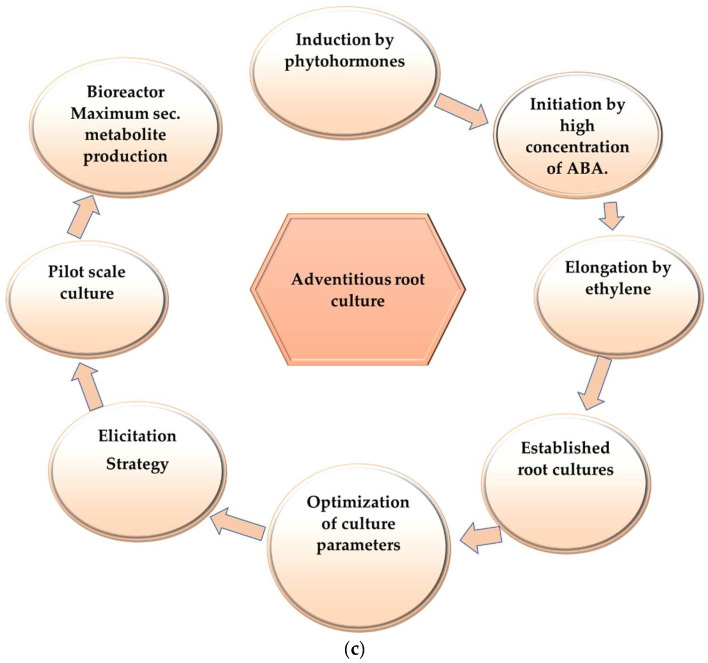
This figure shows a brief comparison between adventitious and hairy root culture and the process of their induction: (**a**) comparison between hairy root culture and adventitious root culture; (**b**) flowchart of hairy root induction; (**c**) flowchart of adventitious root culture.

Moreover, hairy root cultures produce complex structures, i.e., opine-like substrates which are toxic to mammalian cells, and due to the complexity of these structures, hairy roots are difficult to use as a crude extract, because of the high purification cost for opine-like structures [24]. Adventitious root culture is free from opine-like complex, toxic substrates, and provides the system to study the coordination between primary and secondary metabolism [25]. Figure 1 represents a brief comparison between adventitious and hairy root cultures and the process of their induction.

## 5. Media Properties and Culture Condition Effects

Different factors influence the optimal growth of adventitious root culture of medicinal plants which include the type and strength of the media, the concentration of carbon source, pH, and inoculum density. Various culture media have been used to optimize nutrient requirements [26]. Different types of media have different effects on the growth and development of adventitious roots grown in vitro to produce secondary metabolites. Furthermore, different properties of media such as media type, salt strength, sucrose composition, and pH have been studied in terms of the adventitious root culture of medicinal plants.

Ref. [27] reported that different culture media have different effects on induction and proliferation of adventitious root culture. Murashige and Skoog (MS) media were very effective for this purpose in culturing many medicinal plants [28,29]. The optimum conditions were achieved by changing concentrations of MS media, for example, full MS media was used for *Boesenbergia rotunda* and *Vernonia amygdalina*, whereas half MS medium was used for *Camellia sinensis* and *Echinacea purpurea* [30,31]. So, the most suitable culture media for the growth of the adventitious root of medicinal plants are greatly controlled by the plant species itself [32].

In addition to the media used, a different concentration of carbon sources is utilized by different species of medicinal plants, which shows that proper concentration of sucrose can induce the optimum growth of adventitious root biomass [33]. Ref. [27] reported that suitable concentrations of sucrose also increase the production of secondary metabolites. Osmotic agents like sorbitol and mannitol also affect the root dry weight and root growth was increased at 0.1 M mannitol or sorbitol concentration [34]. pH is another factor that strongly influences plant tissue culture increasing biomass and secondary metabolites accumulation. Ref. [34] reported that root growth was at a higher peak at a pH range from 6.0 to 6.5 and strongly inhibited at pH below 4.0 or above 7.0.

## 6. Role of Plant Growth Regulator

The rooting process can be stimulated by different hormones provided artificially and its physiological stages can be affected by changing the auxins concentration inside the roots [35]. Auxin, a plant hormone that comes from the amino acid tryptophan, controls many functions in cellular processes like growth, development, and proliferation of plant cells. The most widely used and naturally occurring auxins are indoleacetic acid (IAA) and indole-3-butyric acid (IBA). Along with natural auxins, different synthetic auxins have been synthesized, for example, 1-Naphthalene acetic acid (NAA) and 2,4-Dicholorophenoxacetic acid (2,4-D) [36].

Different types of auxins have different effects on the process of induction and proliferation of adventitious root culture. Of them, IBA is the most efficient type of auxins for the induction and proliferation of the adventitious root culture of medicinal plants [36]. The importance of auxins in root culture has been reported by many investigators. Auxin treatment strongly affects the root growth and depending on the concentration used, auxins either stimulate or inhibit the root production [37].

To determine the effect of growth regulators (auxins), adventitious roots were grown under different concentrations of indole-3-butyric acid (IBA) and 1-Naphthaleneacetic acid (NAA). By increasing the concentration of both IBA and NAA, root weight was increased and IBA was very effective as compared to NAA [38]. A higher concentration of auxins also decreases the rooting efficiency, because at high concentration auxins may become herbicides which prevent the induction of adventitious roots from explants [39].

Some investigators have reported that the effect of growth regulators on adventitious root growth differs from species to species. For example, [40] reported that in *Gymnema* *sylvestre* cells, NAA was very efficient as compared to IBA. Meanwhile, [41] reported that the dry mass produced from adventitious roots of *Echinacea aungustifolia* was the highest at IBA than the dry mass produced by NAA. Similarly, IBA was found more effective in adventitious root cultures of *Panax ginseng* compared to NAA [42]. Media that contains a large concentration of NAA encourage the formation of callus-like mass bodies characterized by short roots which produce low dry mass, whereas IBA is the most effective in induction and elongation of roots in *Karwinskia calderonii* root culture [43].

## 7. Optimization Strategies to Improve Secondary Metabolite Production

**Figure 2:**
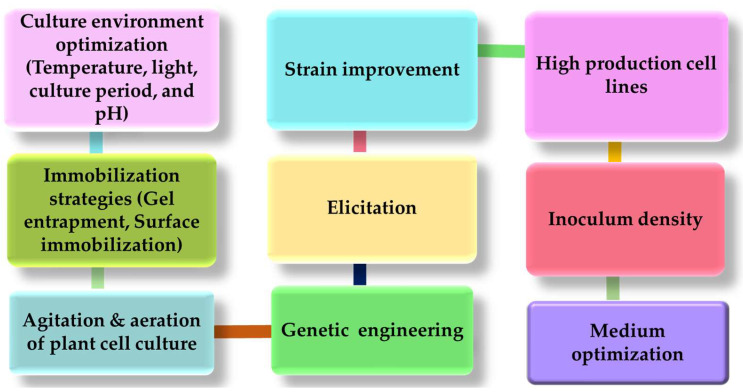
Optimization strategies to improve secondary metabolite production.

Recently, the production of secondary metabolites by using plant cell and tissue culture has become exciting new science. Several approaches are being routinely used by plant biotechnologists to improve secondary metabolite production by the selection of high producing cell lines, strain improvement, medium optimization, metabolic engineering, and elicitation as presented in Figure 2. One of the most important requirements is the in-depth knowledge of biosynthetic routes and mechanisms involved in the accumulation of secondary metabolites. The lack of knowledge of underlying mechanisms involved in the biosynthesis of useful secondary metabolites is the first barrier in developing commercial processes. One of the biggest challenges in the industrial application of plant cell, tissue, or organ culture for the efficient production of commercially relevant bioactive compounds has been stated to have a low product yield [44].

Several factors (chemical or physical) are being explored that may influence the production of secondary metabolites. Growth hormones often play a pivotal role in secondary metabolite production and accumulation in plants. For example, high auxin levels increase the growth of adventitious roots but may have a toxic effect on desired product formation. However, hairy root cultures induced by *Agrobacterium rhizogenes* tend to proliferate without the help of plant growth regulators. Other problems related to plant cell culture are their slow growth rates, high shear sensitivity, aggregates formation, and large size compared to microbial cells are responsible for a decrease in research and industrial exploitation.

Numerous approaches are being exploited that lead to the enhancement of secondary metabolite production in plants. Cell lines obtained from high-producing parent plants will also produce an elevated level of desired metabolites. The selection of storage or production cells is highly important as variable levels of production have been shown by different plant cells. Environmental factors such as sunlight, heat, pH, and nutrient levels can also affect the level of secondary metabolite production. For example, the normal induction temperature range for callus culture is 17–25 °C, although each plant species may have a different optimum temperature. The extreme high and low pH can be toxic to plants hence medium pH is generally adjusted between 5–6 [45].

The intensity, quantity, and irradiation period of light have a considerable effect on plant growth and the production of secondary metabolites. Moderate light intensity has been shown to have a stimulatory effect on the production of some secondary metabolites like artemisinin in *Artemisia annua*, flavonoids [46], in *Petroselinum hortense*, and anthocyanins in *Centaurea cyanus* [47]. In some species, light is seen to have an inhibitory effect on the production of secondary metabolites such as monoterpenes in *Citrus limon* [48].

The growth, proliferation, and secondary metabolite accumulation of plant cells, tissues, and organ cultures can be affected by inoculum density. Relatively high inoculum density is used to initiate the cell suspension cultures because low densities often fail to initiate the growth. The inoculum size depends on the nutrient composition of the medium, the cell line, and other culture conditions. Medium conditioning is also a factor that can be used to minimize the inoculum density, but it has gained not much attention [49].

It has been suggested that developing special modeling systems can help researchers explore the growth features of in vitro cultures as well as the impact of culture conditions. In plant suspension cultures, the choice of culture medium, the use of appropriate nutrients, their ideal concentrations, and environmental conditions can all help to increase the yield and productivity of desired metabolites. The medium composition can be a crucial factor affecting metabolism and cell physiology. The most used medium for cell suspension culture is Gamborg’s (B5), Linsmaier and Skoog (LS), and Murashige and Skoog (MS). However, it is critical to determine the optimum nutrient content and evaluate the impact of selected medium components on growth and product synthesis, as well as strike a balance. This is especially important for plant secondary metabolism because in some circumstances that are appropriate for plant growth can have a deleterious effect on the accumulation of desired product, and vice versa [50].

Genetic engineering is the combination of characteristics otherwise present in different plants and different cells in a single organism and the introduction of specified and active regulation mechanisms [45]. Metabolic engineering in plants can be achieved by elucidating the various pathways and genes encoding the regulatory proteins or metabolic enzymes to increase the production of the desired metabolite. Secondary metabolite accumulation can also be enhanced by blocking the unnecessary and competitive pathways [51].

The capacity of cultured plant cells to convert exogenously provided substances offers extensive potential towards the remodeling of natural and synthetic compounds. The capacity of cultured plants cells to enzymatically alter the molecules can be used in bioconversion processes. In plants, there are separate compartments for the synthesis and accumulation of secondary metabolites. The introduction of an additional artificial site for the storage of secondary metabolites can be a practical tool to enhance total metabolic production. Inhibition of negative feedback can also boost the accumulation of the desired metabolite.

Immobilization strategies can scale up the production of secondary metabolites by persistent process operation. Immobilization can easily overcome the problems associated with plant cell cultures such as low shear resistance and aggregate formation. With the advancements in immobilization techniques, a substantial increase has been seen in the number of applications of valuable secondary metabolites [52]. Gel entrapment and surface immobilization are the most used methods for cell immobilization in plant cells. The most widely used matrix in gel entrapment is gelation, agarose, calcium alginate, and polyacrylamide. The low shear sensitivity increases in secondary metabolite production, ease in downstream processing, low risk of contamination, and reduced cost are some of the major advantages associated with the immobilization of plant cells [53]. Agitation and aeration of plant cell cultures in large-scale bioreactors also play a crucial role in growth and secondary metabolite production. Although the agitation accelerates the culture growth by mass transfer, high shear stress can have harmful effects on cells viability. The shear sensitivity of plant cells restricts the use of high agitation speed that can otherwise prevent aggregate formation. Stirred tank bioreactor and bubble column bioreactors are most used for plant cell cultures to overcome rheological problems [25]. Aeration plays a significant role in oxygen transfer and maintenance of aerobic conditions in plant cell cultures. Although the oxygen need of plant cells is low due to their slow respiratory rates, oxygen supply can have a significant effect on the secondary metabolite accumulation as well as the growth of the plant [54].

## 8. Elicitation

The basis of elicitation lies in plant defense mechanisms. Any threat posed to plant either by pathogenic attack or biotic and abiotic stress will result in secondary metabolite production. Any factor either environmental or biological that has the ability to induce secondary metabolism in the plant is known as an elicitor [55].

Elicitors are compounds from various biotic or abiotic sources that can activate signal transduction pathways, which may enhance secondary metabolism in plant cells. Signal detection of the elicitor by a cell surface receptor is required for elicitor-mediated defensive responses [55]. Following that, some significant cellular and molecular events are transduced, culminating in the stimulation of gene expressions that lead to the synthesis of numerous proteins related to plant defensive responses as well as secondary metabolite synthesis and accumulation [55]. For instance, low molecular weight antimicrobial compounds are produced because of elicitation posed by pathogenic microbes.

Elicitors can be exogenous or endogenous factors that can trigger the production of secondary metabolites. The endogenous or biological elicitors include glycoproteins, organic acids, and membrane polysaccharides such as cellulose, pectin, glucans, chitosan, etc., while the exogenous Elicitors consist of heavy metals, pH and temperature, intensity and duration of light, and nutrients in culture medium. The secondary metabolism in plants can be induced by physical components such as osmotic shock, water stress, ultraviolet (UV) radiation and ozone, etc. [56]. Upon elicitation, by any of these compounds a cascade of reactions is originated, that activates the plant’s innate immune system. To mediate the defense response, the plant signaling compounds such as nitric oxide, salicylic acid, and methyl jasmonate play an important role.

The mechanism of elicitation involves thousands of intertwined events that make it very complex. The elicitation mechanism varies in accordance with the available nutrients, stages of the cell cycle, type and concentration of elicitor, and physicochemical environment of the plant [57]. The first step of elicitation is signal recognition by binding of elicitor to its corresponding receptor on the plasma membrane. This will initiate the cascade of events such as ion flux, ROS outburst, G protein activation, etc. [58]. The activated/phosphorylated proteins will eventually stimulate the expression of genes that are involved in the pathways of secondary metabolites production [59].

**Figure 3:**
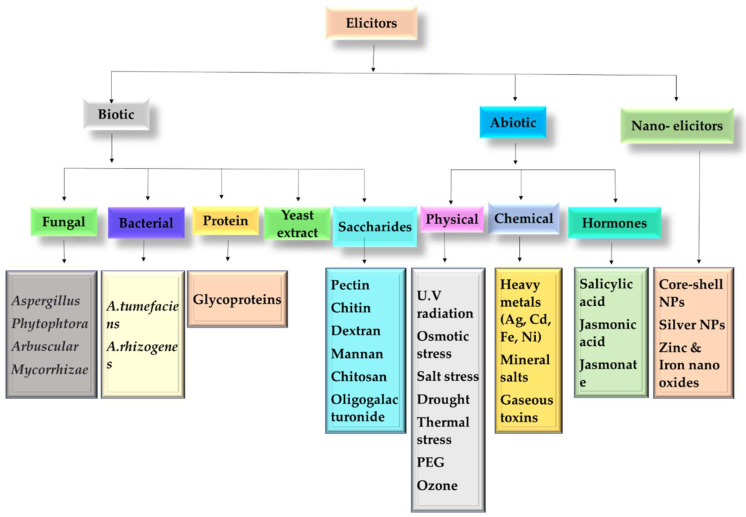
Elicitation, as a powerful means to improve the production of high-valued compounds.

Jasmonic acid (JA)-induced production of terpenoid indole alkaloids is one of the best-studied examples of elicitor-mediated secondary metabolite induction in hairy root culture [60]. The JA and related chemicals also serve as elicitor signal transducers for the manufacture of a variety of secondary metabolites, including alkaloids. For alkaloid biosynthesis, where jasmonates work by coordinating the expression of several biosynthetic genes, the effect of jasmonates on secondary metabolism has been examined in depth. Figure 3 represents the different types of elicitors used as a powerful means to improve the production of high-value compounds.

### 8.1. Biotic Elicitors

There are various compounds that originate from living organisms either bacteria, viruses, and fungi, or from the plant itself that can enhance the production of secondary metabolites are known as biotic elicitors. The most used biotic elicitors are proteins, carbohydrates, fungi and plant growth-promoting bacteria, etc. [61]. The proteins enhance the secondary metabolite production by modulating the regulation of ion fluxes on plasma membrane ion channels in response to some external stimuli. For example, in plant cell cultures glycoprotein can enhance the production of phytoalexin. Carbohydrates have been associated with the elicitation of many secondary metabolites such as phytoalexins accumulation in cotyledons can be enhanced by Oligogalacturonides (OGAs) [62]. Plant growth-promoting rhizobacteria (PGPR) are also known to stimulate to biosynthetic pathways of many useful secondary metabolites such as jasmonic acid in plants [63]. Salicylic acid and jasmonic acid are the most common plant hormone that is used as elicitors in plants.

### 8.2. Abiotic Elicitors

The physical and chemical elicitors that have non-biological origins are known as abiotic elicitors. Abiotic elicitors have been successfully used at different growth stages to stimulate the production of plant phytochemicals. The most employed abiotic elicitors include temperature stress, heavy metals, light intensity, salinity, drought, etc. Heavy metals such as Ag, Cd, Fe, and Ni can stimulate the accumulation of secondary metabolites by modulating their biosynthetic pathways [64]. Light has a stimulatory effect on many secondary metabolites production such as zingiberene and gingerol in *Zingiber Officinale* [65]. The field-grown plant also showed an increased accumulation of phenolic compounds and essential oils when exposed to UV radiations [66]. Elevated temperature can cause leaf senescence while increasing the secondary metabolite production in the roots of *Panax quinquefolius*. In some plants, cold temperatures can elicit secondary metabolite production such as sorbitol, inositol, raffinose, and saccharose are produced as cryoprotectant compounds in extreme winters [67]. Water deficiency or drought is a type of physical elicitor that can increase the secondary metabolites production in plants by modulating their biochemical and physiological properties [68]. Salicylic acid is known to stimulate the expression of defense genes and thus increase plant resistance against diseased pathogens [69], while jasmonic acid acts as an effective elicitor for the synthesis of many useful secondary metabolites such as flavonoids, terpenes, and alkaloids, etc.

### 8.3. Nano-Elicitors

Recently, nanoparticles are being explored as an emerging new class of elicitors. Some of the recent studies have shown the promising potential of nano elicitors in the induction of secondary metabolites accumulation in plant cell cultures. For example, elicitation with core–shell silver nanoparticles has shown an increased production of artemisinin in the hairy root culture of *Artemisia annua* [70]. A decrease in anthocyanin and flavonoid contents and an increase in carotenoid and saponin contents were shown in *Calendula Officinalis* upon elicitation by silver nanoparticles [71]. Zn and Fe nano-oxides have been reported to stimulate hypericin and hyperforin production in cell suspension cultures of *H. perforatum* [72]. Along with the use of metallic nanoparticles as nano elicitors, biotic elicitors such as Methyl Jasmonate, jasmonic acid, etc. can be explored as nanoparticles by encapsulating them into biodegradable polymers which can increase the production of secondary metabolites without affecting the growth of the plant [73]. Some studies also reported the increased productivity of nutraceuticals and the nutritional quantity of crops by the applications of nanotechnology. For instance, aloin is the most important secondary metabolite of aloe vera that has many important properties like antibacterial, antifungal, and antimalarial. Elicitation with abiotic nanoparticles NH_4_NO_3_ can increase the production of aloin up to 127% [73].

## 9. Scale-Up of Culture Process

The demand for plant secondary metabolites is increasing day by day due to their vast array of applications in the health and pharmaceutical industry. To achieve this rising demand of the global market, it is necessary to produce them on large scale. The bioreactor technology is the best alternative for growing plants conventionally to scale up the production of plant secondary metabolites [74]. The cultivation of plant cell cultures in large-scale bioreactors is more profitable and feasible as we can control the entire process to produce high-quality yields in bulk quantities [74].

The primary challenge in commercializing plant-derived secondary metabolites is scaling up the culture in a large-scale bioreactor. Because when cultures are transferred from shake flasks to bioreactors and then scaled up from pilot to industrial size, the environment in which plant cells and roots are cultivated might be changed. Due to this shifting effect of shear stress, oxygen supply, and gas composition in bioreactors reduced productivity has been observed [74,75].

The area of plant cell fermentation and scaling-up has received a lot of attention in recent decades. Plant cells are currently being cultured in amounts up to 75,000 L, and specialized bioreactor systems and effective plant cell culture systems have been developed for growing plant cells [76]. Despite many reports, only a few commercially effective experiments for the generation of adventitious root biomass and bioactive chemicals at industrial scale reactors have been reported. Ref. [77] scaled-up ginseng adventitious root culture in a 500 L balloon-type bubble bioreactor (BTBB), while [8] scaled up ginseng adventitious root culture in a 10,000 L BTBB.

The induction of adventitious roots from selected explants, as well as the optimization of process parameters, are all involved in scaling up adventitious root culture for the generation of biomass and secondary metabolites. Studies are being carried out on the growth kinetics and exploring suitable techniques for higher metabolite accumulation without affecting root growth, and cultivation of adventitious roots in pilot-scale bioreactors. High sparge rates may remove carbon dioxide and other nutrients from the culture media, depending on the metabolic activity of the cells. In these instances, the air-lift bioreactor is the most suitable reactor choice to effectively culture the suspension cells or roots [78].

## 10. Case Studies

### 10.1. Polygonum multiflorum

*Polygonum multiflorum* Thunb. (Polygonaceae), also known in the East as “He-Shou-Wu” and in the West as “Fo-ti”, is one of the most important and commonly used traditional Chinese medicines (TCM) in therapeutic practice [79]. It is a popular Southeast Asian medicinal herb with high levels of phenolic components such as stilbenes, flavonoids, tannin, anthraquinones, and phospholipids, which are responsible for its pharmacological and antioxidant activities [80,81]. In China, Korea, and Japan, the root extracts of this herb were traditionally used as a hair dye, a liver and kidney tonic, and an anti-aging agent [82]. The plant has been used to treat coronary heart disease, hyperlipidemia, and neurosis, as well as aging-related disorders [83].

The increased demand for this plant has recently resulted in overexploitation of natural habitat yet; the bioactive components in *Polygonum multiflorum* roots are unstable in the natural setting. Field production has several limitations, including low yields, slow growth cycles, and quality and quantity changes caused by severe environmental, seasonal, and geographic factors [81]. Apart from advancements in plant cell culture, recent results in large-scale bioreactor synthesis of various plant secondary metabolites using adventitious root culture appear to be a promising strategy [8].

As a result, adventitious root cultures in bioreactors are recognized for their rapid growth and stable metabolic profiles that are comparable to those of field-grown plants [84,85]. In a 500 L pilot-scale bioreactor, an optimal yield of 4.01 kg dry root biomass and 287.12 mg/L total phenolic productivity of *Polygonum multiflorum* was attained [86]. In the study, [87] investigated the effects of several auxins on *Polygonum multiflorum Thunb*. Adventitious root cultures in a 3 L balloon-type bubble bioreactor (BTBB). IBA (1, 2, and 4 mg/L) promoted root development more effectively than NAA. Low MS salt strength (0.25 and 0.5X MS) enhanced total phenolics and flavonoids accumulation but decreased biomass accumulation. The maximum root biomass was obtained after four weeks of culture in full-strength MS medium supplemented with 2 mg/L IBA and 5% sucrose [98.46 g/L fresh weight (FW); 13.46 g/L (dry weight) and the accumulation of bioactive substances (total phenolic compounds, 53.08 mg/g dry weight; total flavonoids, 25.10 mg/g dry weight).

The effects of abiotic (methyl jasmonate) and biotic (yeast extract and chitosan) elicitors on adventitious root cultures of *Polygonum multiflorum* for improving bioactive component synthesis were examined. In comparison to the control, yeast extract resulted in considerably increased dry root biomass (9.98 g/L) and relative growth rate (*p* ≤ 0.05). Abiotic elicitor-treated cultures had a larger percentage of dry weight than the other samples. MeJA had much better elicitation efficiency than the other treatments, according to HPLC analysis of several bioactive compounds. Under 50 molar MeJA treatments root dry weight increased by almost 2-fold (22.08 mg/g dry weight) compared to the control (10.35 mg/g dry weight) [88].

Hairy root culture is another choice for the enhanced production of secondary metabolites. At 20 days, hairy roots cultured on MS liquid medium supplemented with 30 g/L sucrose showed the maximum accumulation of biomass (99.05 g/L FW [fresh weight] and 10.95 g/L dry weight) as well as the highest production of anthraquinones (emodin 211.32 µg/g dry weight and physcion 353.23 µg/g dry weight). When compared to the untransformed control root, suspension cultures produced a nearly 9.5-fold increase in biomass after 20 days of culture, while hairy root biomass produced in suspension cultures had 3.7- and 3.5-fold higher levels of emodin and physcion, respectively [89]. Hairy root culture of *Polygonum multiflorum* produced root biomass (105.2 g/L of FW, 9.7 g/L of dry weight), which is 10-fold more than non-transgenic roots. It also increased total phenolic content (26.64 mg/g dry weight), whereas non-transgenic roots accumulated 8.36 mg/g dry weight. Following exposure to 50 molar methyl jasmonate for 5 days the level of phenolic compounds in HRCs increased by more than 2.5-fold.

Ref. [90], used co-cultivation of plant material with *Agrobacterium rhizogenes* strain LBA942 to induce transformed roots (hairy root) in *Polygonum multiflorum* and investigated the effects of exogenous phytohormones on hairy root growth and anthraquinone biosynthesis in hairy root suspension cultures. They discovered that 2,4-D inhibited hairy root growth and anthraquinone biosynthesis and that NAA and BAP supplementation aided hairy root growth and anthraquinone accumulation. To produce hairy roots in *P. multiflorum*, *A. rhizogenes* R1601 was used. Murashige Skoog (MS), 1/2 MS and B5, were tested to see which combination was best for the culture medium. For hairy root growth, MS medium was the best of the four media. Meanwhile, the hairy roots growth kinetics and nutrient consumption findings revealed that they had a sigmoidal curve and that the best inoculation period for them was 18–21 days. The analysis of the anthraquinone component shown that the hairy roots had a rhein concentration of 2.495 g/g, which was 2.55 times greater than that of natural plants [91].

The above study suggests that adventitious root culture in a bioreactor system with an elicitor is a good alternative strategy to hairy root culture for large-scale production of *P. multiflorum* adventitious roots with high biomass and bioactive compounds accumulation.

### 10.2. Withania somnifera

*Withania somnifera* (L.) Dunal, commonly known as ashwagandha, Indian ginseng, or winter cherry, is a tomentose evergreen shrub of the family Solanaceae [92]. It is widely distributed in Asia, Africa, the Mediterranean region, and the Middle East, apart from India [93]. More than 200 primary and secondary metabolic components of *withania somnifera* have been studied in multiple investigations. The importance of *Withania* in the medicinal world has been acknowledged due to its maximum accumulation and variety of forms of withanolides. Researchers became interested in all the known withanolides variants because of their health-promoting activities [94,95,96,97,98,99].

Major Withanolides found in this species, such as withanolide A and withaferin A, have been shown to exhibit distinct anti-carcinogenic, anti-Parkinson’s, and anti- Alzheimer’s properties [100,101]. Gynecological diseases, bronchitis, arthritis, rheumatism, and drug addiction, such as opiate addiction, are also treated with *Withania somnifera* [95,102].

Field-grown plant materials have traditionally been utilized for commercial withanolides synthesis, although genotype and environmental circumstances have a significant impact on product quality and phytochemical constituent levels [103,104]. Field cultivation is time-consuming and labor-intensive, and it cannot supply the global demand for Ashwagandha. Transferring these roots to suspension culture and mass propagating them in bioreactors reduces the time gap between them and field-grown roots while also ensuring high quality [105].

Plant cell and organ cultures are potential methods for obtaining important metabolites specific to plants [51]. In vitro root culture of *Withania somnifera* has already been attempted in a few cases [106]. Within five weeks, adventitious roots cultivated in flasks with half-strength MS liquid medium containing 0.5 mg/L IBA and 30 g/L accumulated more biomass (108.48 g/L fresh weight and 10.76 g/L dry weight) and had a higher withanolide-A content (8.8 0.20 mg/g dry weight). In suspension cultures, there was a nearly 11-fold increase in fresh biomass, and adventitious root biomass produced in suspension cultures had a 21-fold higher withanolide A content when compared to the leaves of wild plants [104]. After treating root biomass (11.70 g FW) with 150 µM SA for a 4-h elicitor exposure period, 64.65 mg/g dry weight withanolide A (48-fold), 33.74 mg/g dry weight withanolide B (29-fold), 17.47 mg/g dry weight withaferin A (20-fold), 42.88 mg/g dry weight withanone (37-fold), 5.34 mg-1 dry weight 12-deoxy withastramonolide (ninefold), When compared to untreated cultures, 7.23 mg/g dry weight withanoside V (sevenfold) and 9.45 mg/g dry weight withanoside IV (ninefold) were obtained after 10 days of elicitation (40th day of culture) [107].

The influence of carbon source and initial medium pH on the growth and synthesis of withanolide A in *Withania somnifera* adventitious root cultures was examined. A 2% sucrose concentration was shown to be optimum for both biomass (113.58 g/L FW and 11.33 g/L dry weight) and secondary metabolite accumulation (8.93 mg/g dry weight). The biomass of adventitious roots was best when the initial medium pH was 5.8 (113.26 g/L FW and 11.33 g/L dry weight), whereas withanolide A production was highest when the medium pH was 5.5 (9.09 mg/g dry weight) [108].

For secondary adventitious root proliferation, primary adventitious roots with an inoculum mass of 15 g FW were cultivated for 6 weeks in the same medium. Aluminum chloride at 10 mg/L was used as an abiotic elicitor at the end of a 4-week culture with a 4-h exposure time, which increased withanolides productivity. When compared to aluminum chloride treatment under similar growth conditions, the biotic elicitor chitosan at 100 mg/L stimulated higher production of all withanolides [107].

In hairy root culture of *Withania somnifera,* [108] reported 3 percent sucrose to be optimum for biomass accumulation (11.92 g/L dry weight) while 4 percent sucrose stimulated the formation of withanolide A (13.28 mg/g dry weight). Hairy root biomass was maximum at pH 5.8 (12.1 g/L dry weight) and withanolide A production was highest at pH 6.0. Hairy root induction was used on transformed *Withania Somnifera* Ws-Sgtl4 leaf explants, and biomass accumulation was measured. Salicylic acid and methyl jasmonate were used to examine Ws-Sgtl4 gene expression at various time exposures.

The findings revealed that Ws Sgtl4 gene expression increased overall withanolide yield and withanolides-A synthesis in *Withania somnifera* hairy root culture in response to elicitors [109]. After 30 days of culture, hairy roots with an initial inoculum mass of 5 g FW were elicited separately with methyl jasmonate and salicylic acid (SA) at various concentrations for varying exposure durations. From 40 day-old harvested hairy roots elicited with 150 µM SA for 4 h exposure time, increased production of biomass (32.68 g FW and 5.54 g dry weight; 1.23-fold higher), withanolide A (132.44 mg/g dry weight; 58-fold higher), withanone (84.35 mg/g dry weight; 46-fold higher), and withaferin A (70.72 mg/g dry weight; 42-fold higher) were obtained [110]. The hairy roots were elicited with natural polysaccharides of sodium alginate (SA), k-carrageenan (kC), and chitosan (CH) at varied concentrations, which resulted in a large amount of WA compound in majority of cases. Among the various elicitors, 100 mg/LCH resulted in a 4.03-fold increase in WA production as compared to the control. This is the first time that natural polysaccharides have been employed as elicitors in *Withania somnifera* hairy roots [111].

According to above mention study total withanolides contents in transgenic hairy roots of *Withania somnifera* were higher than the non-transformed roots. Moreover, treatment with elicitors such as SA and MeJA were discovered to increase the accumulation of withanolides in hairy roots, so it is preferred over adventitious root culture for large-scale production of secondary metabolites in *Withania somnifera.*

### 10.3. Echinacea purpurea

*Echinacea purpurea*, also known as purple coneflower, has been acknowledged globally owing to its increasing medicinal worth. The main bioactive compounds reported in *Echinacea* extracts are phenolics, alkylamides, and polysaccharide/glycoproteins. The phenolics majorly include Echinacoside, cynarin, cichoric acid, caftaric acid, and chlorogenic acids (CADs), Of these, cichoric acid is the center of attention as it displays immunostimulatory properties and is shown to promote phagocytosis both in vitro and in vivo. It has also been reported to possess antiviral activity prominently against HIV [112].

*E. purpurea* extracts have been reported to be effective against various viruses, respiratory tract infections, inflammatory disorders, and skin diseases like atopic eczema [113]. Furthermore, in vitro studies suggested that *E. purpurea* extracts possessed high antioxidant, antidiabetic, and antihypertensive properties [77]. Despite the enormous success of *E. purpurea* on the market, some major challenges pose problems to the industry such as seed dormancy, low germination rates a relatively long maturation time, and fungal pathogens with the substantial challenge being product standardization [19,112].

Conventionally, propagation of *E. purpurea* was done by seeds, crown division, or root cuttings but these processes have restricted propagation potential for producing sizeable numbers of plants, can cause genetic variability, and are time-consuming. In vitro micropropagation techniques, such as adventitious root, hairy root, and somatic embryogenesis, have the potential to produce hundreds of clonal plants from cuttings of a parent plant [21].

The viability of employing mass cultivation of the adventitious roots of *E. purpurea* in balloon-type bubble bioreactors was evaluated to produce caffeic acid derivatives, which are known for their pharmaceutical and therapeutic values. After 4 weeks of culture in BTBB (5 L capacity with 4 L half-strength MS media), an approximately 10-fold increase was reported in biomass and secondary compound. An inoculum density and aeration rate of 7 g/L and 0.1 vvm, respectively, were reported to be best suitable for inducing the accumulation of biomass and secondary metabolites. The concentration of cichoric acid was the highest (26.64 mg/g dry weight) of the three caffeic acid derivatives evaluated [114].

The effects of 24-epibrassinolide (24-eBL) and l-phenylalanine (l-phy) were evaluated on different parameters such as the root growth, total phenolics, total flavonoids, and caffeic acid derivatives (CADs) accumulation in *Agrobacterium rhizogenes* mediated hairy roots of *Echinacea purpurea*. 1.0 mg/L 24-eBL was reported as the optimum concentration, among the 24-eBL applications, which provided the highest contents of total phenolics, total flavonoids, cichoric acid, caftaric acid, Echinacoside, and p-coumaric acid. The results displayed that 24-eBL and l-phy treatments successfully enhanced the production of secondary metabolites in transgenic hairy roots of *E. purpurea* and 24-eBL implementations were considered as a more effective strategy when compared to l-phy [115].

Rhizobium-based transformation efficiency of *Echinacea* *purpurea* is influenced by several factors and therefore, there is room for optimization. For instance, the effectiveness of the utilized bacterial strain is important; R1000 strains were superior for transforming *E. purpurea* with petioles, whereas A4 strains gave the best results with leaf explants [116]. During co-cultivation of agrobacterium with the plant tissue, the addition of inducers to the medium also enhances efficacy. Ref. [87] reported that indole-3-butyric acid increased the production of hairy roots in *E. purpurea* by 14-folds. To improve transformation, other inducers of Rhizobium-associated gene transfer in plants such as 6-benzylaminopurine, 2,4-dichloro phenoxyacetic acid have also been applied to *E. purpurea* hairy root cultures [116]. The use of elicitors such as plant hormones, biotic and abiotic compounds, and physical injury displays promising results in stimulating the production of valuable secondary metabolites [112].

Supplementation of sodium nitroprusside to the growth medium, in the adventitious root culture of *E. Purpurea*, increased the accumulation of flavonoids and CADs [117]. Salicylic acid displayed a twofold increase in cichoric and caftaric acid in flower heads of *E. purpurea* flower heads, and when applied as a foliar spray to field-grown plants, approximately a fourfold increase of CAD in the roots was reported [118]. Biotic elicitors such as yeast extract imitate a pathogenic fungal infection and consequently stimulate the production of phenolics in *Echinacea* *purpurea* [119].

Inspire of these imposing effects on phytochemical content, elicitors are also reported to have an inhibitory effect on growth. For this purpose, a two-phased approach is considered inevitable for the optimization of phytochemical production. Ref. [120] evaluated that addition of methyl jasmonate to the culture on the 28th day, had a 3-fold increase in the Echinacoside content in root cultures of *E. angustifolia*, without reducing the biomass. As the production of both biomass and phytochemicals often decreases at industrial scales, therefore it is better to utilize chemical elicitors in large-scale bioreactors [29].

The basis of tissue culture is growth medium and innumerable studies have evaluated what combination of nutrients provides the finest growing environment for *Echinacea purpurea* cultures. The optimal media may differ, and it mostly depends on the *E. purpurea* species and tissue being cultured. For example, [41] in earlier work, reported that the maximum biomass of adventitious roots of *E. angustifolia* was obtained on a half-strength Murashige Skoog (MS) medium. However, later on, they reported that the maximum biomass of adventitious roots, as well as maximum phenolic content of *E. purpurea*, could be obtained on one-quarter strength Murashige Skoog medium along with 50 g/L sucrose and 1 mg/L IBA [29].

Light treatment is currently reported to alter secondary metabolite formation. The effects of UV-B radiation on *E. purpurea* cultures for short periods in varying exposures were evaluated. All UV-B treatments enhanced caffeic acid and antioxidant activity of callus cells and growth parameters, total phenols content, and antioxidant activity of cell suspensions in a dose-dependent manner. Later on, the same group also tested both types of *E. purpurea* cultures by varying the doses of UV-C and reported similar results [121]. In recent times, ultrasound treatment has been developed as a method of enhancing plant secondary metabolite content, but it has not been considerably tested with *E. purpurea*. Two studies with *E. purpurea* hairy roots grown in bioreactors evaluated that between days 15 and 20 of culture, one 6-min ultrasound treatment at 40 kHz, remarkably enhanced both fresh weight and cichoric acid content over 30 days [21].

Hairy root cultures have several notable properties that are extremely useful for research and industry, which include accelerated growth rate, spontaneous regeneration of shoots, as well as chemical and morphological similarity to the roots of a wild-type plant [122]. Hairy root cultures of all three commercially important *Echinacea purpurea* species are reported to produce high levels of secondary metabolites, namely polysaccharides, alkylamides, CADs, and other phenolics [19,20]. Moreover, transformed roots of *E. purpurea* are genetically stable and possess the ability to maintain a constant production of valuable metabolites over a long period [21]. Furthermore, the rapid growth of *E. purpurea* hairy root cultures on hormone-free media makes them an outstanding means to generate biomass quickly. Due to these characteristics, hairy root cultures of *E. purpurea* are preferred over adventitious root cultures to produce valuable compounds.

### 10.4. Ajuga bracteosa

*Ajuga bracteosa* commonly referred to as Neel-kanthi is a medicinal herb of the family Lamiacea. Traditionally, it has been used as a favorable alternative to the wonder herb cinchona as anti-fungal, stimulant, tonic, and constringent [123]. Recent research has shown that *A. bracteosa* extracts have positive results in the treatment of inflammation, diabetes, malaria, palsy, stomach pain, intestinal dysfunctions, tuberculosis, hypertension, and cancer [124].

The prominent class of secondary metabolites found in different parts of *A. bracteosa* includes phenolic acids, flavonoids, essential oils, tannins, and anolides, etc. [125]. 20-Hydroxyecdysone (20 HE) is an ecdysterone that accumulates in *A. bracteosa* is regarded as a naturally occurring hormone that can control the ecdysis and metamorphosis of arthropods. Furthermore, positive effects of this bioactive compound against human carcinoma cell lines have also been reported [126].

It is getting endangered globally due to devastating harvesting by pharmaceutical industries in its different habitats. Therefore, there is a dire need to protect this medicinal herb by conservation and sustainable utilization. Adventitious root culture is a very appealing technology, among all the other cell culture technologies including hairy root cultures because it has the potential to produce bioactive metabolites in higher quantities, disregarding the environmental constraints in limited time and space [123]

Elicitation strategy is well known to enhance secondary metabolite production. Ref. [123] investigated the effects of two prominent elicitors, methyl jasmonate (MeJA) and phenylacetic acid (PAA) on growth parameters, secondary metabolites production, and antioxidant potential in adventitious root suspension cultures of *A. bracteosa*. The results showed a considerable increase in biomass accumulation, on day 40 of culture as well as the elicitors-induced enhancement in phenolic content, flavonoid content, and antioxidant activity in root suspension cultures of *A. bracteosa*.

Light regulates almost all plant developmental processes. Ref. [123] evaluated the effects of α-naphthalene acetic acid (NAA) in adventitious root cultures of *A. bracteosa* under different spectral lights on different parameters such as growth, secondary metabolism, and biosynthesis of phenolic acid. In AR cultures, grown in presence of blue light, maximum production of polyphenols and flavonoids was recorded. Whereas normal white light and blue light contributed to the highest total protein content of (401.6 μg) and maximum superoxide dismutase (SOD; 2.5 nM) activity respectively. Furthermore, in comparison with other monochromatic lights, red light considerably enhanced the antioxidant potential of the AR cultures.

Ref. [127] for the very first time reported the biotechnologically enhanced production of phytoecdysteroids in *A. bracteosa* both via transformation and elicitation. pRi TR-DNA genes were reported to act as key players in the determination of the morphology of hairy roots. Typical transgenic hairy roots produced more phytoecdysteroids, on the other hand, those with callus-like morphology resulted in enhanced biomass. Elicitation treatment with MeJA for 14 days further increased phytoecdysteroids level.

Other most prominent medicinal plant species for which adventitious root and hairy root culture have been established to produce valuable compounds by employing bioreactors or elicitation strategies are enlisted in Table 1 and Table 2. The increasing demand for natural drug raw materials particularly in the pharmaceutical industry can be fulfilled by these strategies.

**Table 1:** List of adventitious root culture of medicinal plant species to produce valuable compounds by employing bioreactors or elicitation strategy.

Plant	Explant Used	Valuable Compound	Culture Conditions and PGRs	Elicitation Strategy	Increase in Yield of Valuable Compound	Bioreactor Employed	Optimization Strategy of Culture Conditions and Bioreactor	Reference
*Ajuga bracteosa* Wall. ex Benth.	Leaf explants	poly phenols flavonoids	MS media + (1.5) NAA	Different Spectral lights	Enhanced production of polyphenols (44.2 mg) and flavonoids (2.51 mg) were observed in presence of blue light.	_	pH 5.6–5.8 photoperiod (16 h light and 8 h dark) under cool-white light (~100 μmol/m^2^/s) Temperature 25 ± 2 °C	[123]
*Allamanda cathartica* L.	Nodal segment	iridoids	½ MS media + 0.1 μM IAA, 0.5 μM IBA, 1.0 μM NAA	-	Total yield of iridoid glycoside content was highest in S3 sample 5.53 ± 0.03% treated with 0.5 M IBA + 4 percent sucrose+ 120 mM NaCl.	-	Sucrose 3% NACL supplementation Culture period: 4 weeks pH 5.8 relative humidity: 55–60%	[128]
*Angelica gigas* N.	Seeds	decursin & decursinol angelate	MS media + 1.0 mg/L IBA+ 0.6 g/L caseine hydrolysate	yeast extract, chitin, MeJA, SA, and copper	1.5-fold increase in plant yield. 1.7-fold increase in production of decursin.	_	Sucrose: 30 g/L pH: 5.7 Subculturing, every 21 days.	[129]
*Artemisia amygdalina* D.	Leaf explant	Essential oils	MS media + 1.0 mg/L α-naphthalene acetic acid (NAA)	methyl jasmonate	(Me-J: 0.5 mg/L) resulted in the higher production of total phenolic content (3.6 mg), total flavonoid content (2.3 mg), and phenylalanine ammonia-lyase (4.8 U/g× FW).	_	light intensity: ~40 µM/m^2^ s Humidity: 70% Sucrose: 3%	[130]
*Artimisia scoparia* Waldst. and Kit.	Leaf explant	DPPH	MS media + 1.0 mg/L PAA and NAA	MeJA	87% antioxidant potential was achieved in presence of 0.5 mg/L MeJA.	_	Relative humidity: 70% light intensity of ~40 μM/m^2^ s sucrose: 3% pH: 5.8 Culture period: 44 days	[130]
*Asphodeloideae* L.	Young shoots	Aloe-emodin and chrysophanol	MS liquid media with 0.3 mg/L IBA	salicylic acid, methyl jasmonate, and ethephon	Aloe-emodin increased 10–11-fold by SA treatment. Chrysophanol was increased by 5–13-folds by SA treatment.	_	30 g/L sucrose pH 5.8 constant light conditions (light intensity: 7 mE/m^2^ s) culture period: 3 weeks	[31]
*Astragalus membranaceus* L.	Roots	Calycosin-7-O-β-d-glucoside (CG)	MS media + 7 mg/L IBA	MeJA L-phenylalanine	2.02-fold increase in CG content, with 200 µM MeJA for 8 days. 3.12-fold increase in CG by feeding MeJA-elicited CG with l-phenylalanine.	_	Sucrose: 30 g/L PEG treatment	[131]
*Astragalus membranaceus var. mongholicus* F.	Shoot	calycosin-7-O-β-D-glucoside and astragaloside Ⅳ	MS media + 1 mg/L NAA	Green leaf volatiles (hexanal, hexanol, and E-2-hexenal)	Treatments with hexanol and E-2-hexenal significantly increased the content of astragaloside Ⅳ by 81.67% and 81.41%. Treatments with hexanal and hexanol increased the CG content up to 56.07% (0.57 ± 0.05 mg/g) and 65.18% (0.60 ± 0.02 mg/g), respectively	-	Sucrose: 30 g/L Photo period: 16 h light/8 h dark Culture period: 4 weeks	[132]
*Boesenbergia rotunda* L.	Bud explant	pinostrobin	½ MS media + 2.0 mg/L NAA	-	High pinostrobin production (3.54 mg/g) obtained with 50 g/L of sucrose concentration.	-	Sucrose: 50 g/L pH: 5.75 to 5.80 Culture period: 8 weeks	[27]
*Celastrus paniculatus* W.	Leaf explants	Celastrol	MS media + 0.3 mg/L IAA	Silver nanoparticles and acetosyringone	Celastrol increased 1.87-fold by treatment with 10 µg/mL of AgNPs (48 h exposure).	_	3% sucrose pH 5.8 Culture period: 2 weeks	[133]
*Clitoria teretea* L.	Seeds	pentacyclic triterpenoid, saponins, flavonoids, anthocyanins	½ MS media + 2.50 mg/L NAA, 2.50 mg/L (4-chloroindole-3-acetic acid) 4-Cl-IAA	-	Taraxasterol content was detected with peak area 72.01% and 6.35% respectively.	-	Sucrose: 3% Photoperiod: 16/8 h Light intensity: 32.5 µE/m^2^ s Culture period: 6 weeks	[134]
*Codonopsis lanceolata* S.Z.	Leaf explant	flavonoids, total phenolic compound, and DPPH.	MS media + 1.0 mg/L IBA	MeJA and SA	By treatment with 20 µM MeJA, DPPH scavenging activity was 24.2. Flavonoids: 38.45 mg/g of C. lanceolata Phenolic content: 74.53 mg/g	_	Culture period: 5 weeks Sucrose: 30 g/L	[135]
*Curculigo orchioides* G.	Leaf explants	Phenolics and Curculigoside	MS media + 3.0 mg/L NAA in liquid culture	_	Adventitious roots grown in modified ¾ strength of MS medium showed the highest amount of curculigoside (76.521 µg/Treatment).	_	¾ strength of MS medium 4% (*w*/*v*) of sucrose	[136]
*Decalepis salicifolia* (Bedd. ex Hook.f.)	Leaf	Vanillin isomer: 2-hydroxy-4-methoxybenzaldehyde (2H4MB),	WPM liquid media + 0.5 mg/L NAA, 1.0 mg/L Kinetin, 0.3 mg/L IBA	-	The total production of 2H4MB was 4.9-fold higher in adventitious root culture (139.54 μg) as compared to field-grown plants (28.62 μg).	-	Sucrose: 5% pH: 7.0 culture period: 60 days	[128]
*Echinacea purpurea* L.	roots	caffeic acid derivatives caftaric acid, chlorogenic acid, and cichoric acid.	MS media + 2 mg/L IBA	_	A 10-fold increase in biomass and secondary compounds. the concentration of cichoric acid was the highest (26.64 mg/g dry weight)	Balloon-type bubble bioreactor.	Sucrose: 5% inoculum density: 7 g/L FW aeration rate: 0.1 vvm	[114]
*Echinecea pallida* N.		Phenolics, flavonoids	¾ MS media + 1 mg/L IBA	-	ARs were cultured in a 5-L air-lift bioreactor the bioactive compounds (53.5 mg/g phenolics and 37.6 mg/g flavonoids) reached the maximum values, and the productivities of phenolics and flavonoids were 398.7 and 280.4 mg/L.	Air-lift balloon type bioreactors	sucrose: 50 g/L pH: 5.8 nitrogen: 45 mM Phosphorus: 1.56 Mm Culture period: 30 days	[137]
*Eleutherococcus koreanum* M.	Seed-derived plants	eleutherosides B&E, chlorogenic acid, total phenolics, and flavonoids	MS media + 5 mg/L 0.01 mg/L TDZ	MeJA and SA	At 303.93 mg/L of MeJA production of targeted bioactive compounds was 37.77%. High concentrations of MeJA and SA increased DPPH activity and H_2_O_2_ content in the roots.	Airlift bio- reactors	(HN4: NO3-, 5: 25) Sucrose: 30 g/L Gelrite: 2.3 g/L density of AR = 5 g/L aeration volume = 0.1 vvm	[84]
*Eurycoma longifolia* J.	Leaf	Eurycomanone and polysaccharides	¾ MS media + 3 mg/L IBA	-	8.8 mg/Leurycomanone and 2.4 g/L polysaccharides obtained after 40 days of culture in Bubble column bioreactor	Bubble column bioreactor	Sucrose: 40 g/L inoculation density: 5 g/L Aeration rate: 0.05 vvm Culture period: 40 days	[138]
*Fagonia indica* L.	callus explants	Gallic acid Rutin Myricetin Catechin Caffeic acid Apigenin	MS media + (0.5, 1.0 or 2.0 mg/L) IBA, IAA or NAA	MeJA PAA	By 0.5 mg/L MeJA treatment, maximum Total Phenolic Content (TPC; 6.0 mg GAE/g of dry weight) and Total Flavonoid Content (TFC; 5.0 mg QE/g of dry weight) were achieved. Gallic acid (148.0 ± 4.8 μg/mg of dry weight) Rutin (122.3 ± 3.8 μg/mg of dry weight) Apigenin (25.3 ± 0.6 μg/mg of dry weight), Caffeic acid (25.3 ± 0.6 μg/mg of dry weight) and Catechin (9.4 ± 0.07 μg/mg dry weight).	_	Sucrose: 3% MS salts: 0.44% Culture period: 33 days relative humidity: 70% irradiance: 35–45 μ mol/m^2^ s pH: 5.6–5.8	[139]
*Gentiana scabra* B.	Leaf	secoiridoids	MS media + 3.0 mg/L NAA and 0.25 mg/L TDZ	-	HPLC revealed Maximal gentiopicroside (25.59 ± 0.65 mg/g dry weight), swertiamarin (1.61 ± 0.04 mg/g dry weight) and sweroside (4.42 ± 0.11 mg/g dry weight) levels after 4 weeks culture	-	Phytohormones: 30 mg/L α-naphthalene acetic acid and 0.25 mg/L thidiazuron. Photoperiod: 16/8-h Culture period: 8 weeks	[140]
*Ginseng* C.A.Mey. and *Echinacea* L.	roots	ginsenosides and caffeic acid derivatives	MS media + 25 µM IBA	MeJA	Higher production of ginsenosides and caffeic acid derivates was achieved by the establishment of co-cultures with higher inoculum proportion of ginseng to *Echinacea*, ensued by elicitation 200 µM MeJA.	Air-Lift bioreactors	Co-culture system Inoculum proportion of Ginseng to *Echinacea* (4:1 and 3:2) Sucrose: 50 g/L	[83]
*Glycyrrhiza uralensis* DC.	root	Flavonoids glycyrrhizic acid glycyrrhetinic acid polysaccharide	MS media +1 mg/L IBA	10 kDa protein fragments	10 kDa protein fragments increased the Flavonoids, glycyrrhizic acid, glycyrrhetinic acid, and polysaccharide by up to 2.27-fold, 2.64-fold, 2.70-fold, and 2.32-fold, respectively as compared to control roots.	_	Sucrose: 30 g/L Culture period: 35 days	[141]
*Gynura procumbens* L.	Leaf explant	flavonoid	MS media + 5 mg/L IBA	_	Biomass yield of adventitious roots of G. procumbens in temporary immersion bioreactor increased by 5 folds. Isoflavon was detected in adventitious roots at low sucrose treatment. volatile compound and adipic acid were found in all treatments.	Temporary Immersion Bioreactors	various concentrations of sucrose (1, 3, and 5%) various immersion frequency (15 min each 12 h; 5 min each 3 h). Culture period: 21 days	[142]
*Gynura procumbens* L.	Young leaves and internodes	Phenolic compounds and flavonoids	MS media +5 mg/L IBA	_	The greater yield of Biomass (75.38 ± 0.95 g/L), Total phenolic production (27.98 mg/dry weight), and Flavonoid production rates (256.24 mg/dry weight) were achieved from adventitious roots culture in the BTBB.	Balloon-Type Bubble Bioreactor (BTBB).	aeration rate: 0.15 vvm. inoculum density 3 g/L Sucrose: 30 g/L Culture period: 28 days.	[143]
*Hybanthus enneaspermus* L.	Leaf	L-Dopa	MS media + 0.5 mg/L Indole-3-butyric acid (IBA)	SA, Yeast extract, MeJA, AgNO3	Among the different elicitors tested, exposure to SA at 100 µM dosage for 6 h enhanced L-dopa yield 12.64 mg/g dry weight (dry weight) when compared to control culture	-	sucrose: 3% pH: 5.8 exposure times (2–8 h) elicitation period: 6 h culture period: 30 days	[144]
*Hypericum perforatum* L.	roots	Naphthodianthrone derivatives	MS media + 1 mg/L of IBA	Different radiation treatment	hypericins production was enhanced by red light. four-weeks grown roots treated with one-week blue light was an effective stimulator for increasing total phenolic compounds and hypericins.	-	Sucrose: 30 g/L the photon flux density of 50 µmol/m^2^ s	[145]
*Morinda coreia* Buch.-Ham.	Leaf	Anthraquinones and phenolic compounds	½ MS media+ 1.0 mg/L of Indole-3 butyric acid (IBA)	chitosan	On treatment with 0.4 mg/mL chitosan amount of anthraquinones (292.038 mg/g dry weight) and phenolics (86.8 mg/g dry weight) increased till 4th day of the elicitation	-	Two-phase and two-stage culture system ½ MS media Sucrose: (1.5%). chitosan (0.2, 0.4 and 0.8 mg/mL), growth ratio (5.082), fresh weight (1.568 g) and dry weight (0.163 g) of AR were recorded maximum with the concentration of 1.0 mg/L IBA.	[146]
*Oldenlandia umbellate* L.	shoots	Anthraquinones (AQ)	MS media+ 7.5 μM IBA 1 μM IAA	yeast extract, pectin, xylan, α-keto glutaric acid and L- phenylalanine and piroxicam	Treatment with 50 mg/L pectin, resulted in 2.19-fold increase in AQ production.	_	Culture period: 60 days Sucrose: 3% pH: 5.8	[147]
*Oplopanax elatus* N.	Seeds	flavonoids and anthraquinone	MS Media+ 5 mg/L IBA	MeJA	At 200 µM, MeJA significantly increased the contents of quercetin, aloe-emodin, rhein, and emodin, while 225 µM was the optimal concentration for kaempferide accumulation.	Air-lift balloon type bioreactor	Sucrose: 30 g/L pH: 5.8 Culture period: 30 days	[148,149]
*Panax gingseng* C.A.Mey.	Root	Ginsenosides	MS media+ 5 mg/L indole butyric acid	Fungal elicitor	The maximum ginsenoside content reached 29.6 mg/g dry weight when 30-day-old ARs were treated with 200 mg/L fungal elicitor for 8 days	Balloon-type Air-lift bioreactor	Sucrose: 30 g/L Culture period: 30 days 200 mg/L fungal elicitor was selected to treat 30-day-old ARs for 2, 4, 6, 8, and 10 days.	[150]
*Panax quinquefolius* L.	Root	Ginsenosides	MS media + 1 mg/L 2, 4-D, 0.25 mg/L kinetin	Pathogenic fungal elicitors	The maximum ginsenoside production (276.0 mg/L) was achieved with the A. panax (4 mg/L) extract.	Balloon-type airlift bioreactor	sucrose: 50 g/L pH: 5.8 ¾ MS medium supplemented with 5 mg/L IBA air volume: 100 mL/min Culture period: 30 days Elicitation period: 8 days	[90]
*Panax vietnamensis* Ha et Grushv.	Leaf	saponins	Modified MS Media + 5 and 7 mg/L IBA +0.5 or 1 mg/L of single BA, Kin and TDZ	JA, SA, YA and Chitosan (CHN)	Saponins maximum productivity was observed in 150 mg/L YE.	Bubble bioreactor	Sucrose: 30 g/L Ratio of NH_4_^+^NO_3_ 7.19: 18.50 mM/mM pH: 5.8 culture period of 56 days	[151]
*Perovskia abrotanoides* Karel.	Young leaves	Cryptotanshinone tanshinone IIA	MS media + 2 mg/L (NAA)	yeast extract (YE), (MeJA), AgNO_3_, and sorbitol	Increased cryptotanshinone and tanshinone IIA production was achieved with 200 mg/L YE and 25 µM AgNO_3_	_	3% sucrose pH 5.8 ± 0.1 Culture period: 3 weeks (dark)	[152]
*Plumbago indica* L.	Young leaf	plumbagin	Gamborg’s B5 liquid media + 0.1 mg/L NAA	Chitosan + Diaion^®^HP-20 addition	Plumagin increased upto 6.6-fold by chitosan treatment for 72 h. The sequential addition of Diaion^®^HP-20 (10 g/L) to the root cultures after the chitosan treatment for 48 h increased the plumbagin production up to 19.93 mg/gdry weight, which was 1.2- and 10-fold higher than the chitosan treated and untreated root cultures respectively.	-	Sucrose: 20 g/L chitosan concentration: 150 mg/L optimal contact period: 72 h Culture period: 20 days	[153]
*Plumbago rosea* L.	Leaf explants	Plumbagin	MS media + 1.5 mg/L IAA + 1 mg/L IBA	Jasmonic acid yeast extract and sodium salicylate	50 µM jasmonic acid for three days increased plumbagin content in roots to 1.23% dry weight.	_	3/4th strength MS liquid media Sucrose: 30% Root inoculum: 2 g/L	[154]
*Polygonum* *Multiflorum* Thunb.	Leaf	Phenolics and flavonoids	Full-strength MS media + 2 mg/L	Methyl jasmonate (MeJA) and salicylic acid (SA)	Total phenolic compounds increased by 53.08 mg·g^−1^ dry weight and total flavonoids increased by 25.10 mg/g dry weight	Air lift bioreactor	sucrose: 5% Culture period: 4 weeks Aeration rate: 0.1 vvm (air volume flow per unit of culture volume per minute)	[87]
*Prunella vulgaris* L.	Leaf	Phenolics and flavonoids	MS + 0.5 mg/L NAA	-	Higher TPC (0.995 GAE mg/g-DRB) and TFC (6.615 RE mg/g- DRB) were observed in 0.5 mg/L NAA treated cultures.	-	sucrose: 30 g/L Photoperiod: 16/8 h Light intensity: 40 mol/m^2^ s Culture period: 49 days	[155]
*Stevia* *rebudiana* (Bertoni) Bertoni	Plantlets	Polyphenolics and Steviol Glycosides	MS media + gibberellic acid (GA3; 0.5, 1.0, 1.5 and 2.0 mg/L) and 0.5 mg/L NAA	Gibberellic acid (GA3)	The highest TFC accumulation was shown by 2.0 mg/L of GA3, as compared to the control culture (4.74 mg QE/g dry weight on day 30 and maximum stevioside content (7.13 mg/g dry weight) w GA3, as compared to the control culture 3.39 mg/g dry weight.	-	2.0 mg/L of GA3 was optimum concentration for maximum biomass biosynthesis (13.12 g/flask) noticed in exponential phase on 27th day of culture. Culture period: 30 days	[156]
*Talinum paniculatum* Ruiz and Pav	shoot	Saponin	MS media + 10 µM IBA	MeJA and SA	By treatment with 0.2 mM MeJA and SA, saponin production increased by 1.5 and 1.3-fold.	_	Culture period: 28 days Sucrose: 30 g/L	[157]
*Talinum paniculatum* Ruiz and Pav	Leaf explants	saponin	MS media + 2 mg/L IBA	_	Saponin content was increased by combination of aeration rate of 0.5 vvm and inoculum density of 1 g/400 mL	Balloon type bubble bioreactor	Aeration rate: 0.25, 0.5 and 0.75 vvm Inoculum density: 0.5, 1, 2 g/400 mL. Culture period: 14 days	[22]
*Tripterygium wilfordii* Hook. f.,	Leaf	celastrol	1/2 MS media + 0.25 mg/L indole-3-butyric Acid and 0.25 mg/L naphthylacetic acid	Methyl jasmonate (MeJA) and salicylic acid (SA)	100 μM MeJA significantly increased celastrol content in adventitious roots to 6321.27 μg/g dry weight	-	Sucrose: 30 g/L Photoperiod: 16/8 h (day/night) Culture period: 6 weeks	[158]
*Withania somnifera* (L.) Dunal	Leaf	Withanolides	½ MS media + 0.5 mg/L IBA,0.1 mg/L IAA	Methyl jasmonate and salicylic acid	150 μM SA for 4 h elicitor exposure period resulted in the increase production of withanolide A (48-fold), B (29-fold), withaferin A (20-fold), withanone (37-fold 12-deoxy withastramonolide (nine-fold), withanoside V (seven-fold), withanoside IV (nine-fold)	-	culture age: 30 days old elicitation period: 6 h Culture period: 50 days. biomass: kolli hills variety maximum fresh weight (11.70 g), dry weight (1.90 g) Cumbum variety maximum fresh weight (11.40 g), dry weight (1.85 g)	[159]

**Table 2:** List of Hairy root culture of medicinal plant species for the production of valuable compounds by employing bioreactors or elicitation strategy.

Plant Species	Strain	Explant	Valuable Compound	Media + PGRs	Elicitation Strategy	Increase in Yield of Valuable Compound	Bioreactor Employed	Optimization Strategy for Culture Conditions and Bioreactor	References
*Ajuga bracteosa* Wall. ex Benth.	A4, LBA-9402 and ARqua1.	Leaves	phytoecdysteroids	MS media no PGRs	MeJA and coronatine (Cor)	In comparison with the unelicited hairy roots, MeJA doubled phytoecdysteriod content i.e., 8356 µg/g after 14 days of Elicitation.	_	Sucrose: 30 g/L Solidifying agent:0.8% phytagel	[127]
*Artemisia annua* L.	LBA 9402	seedlings	artemisinin	MS media with no PGRs	MeJA Fungal elicitors: Alternaria alternate, Curvularia limata, Fusarium solani, and Piriformospora indica	By using P. indica artemisinin production was increased by 1.97 times. By using combination of MeJA and cell homogenate of P.indica artemisnin production was enhanced by 2.44 times.	_	Sucrose: 30 g/L	[160]
*Astragulas membranceus* L.	*Agrobacterium rhizogenes* LBA9402	Leaf	Phytoalexins	MS media with no PGRs	Chitosan	Treatment with 100 mg/L of chitosan increased yields of formononetin and calycosin by 12.45- and 6.17-fold.	_	Elicitor exposure time: 24 h. Chitosan: 100 mg/L Culture period: 34 days	[161]
*Celastrus paniculatus* W.	MTCC532	Leaf explant	Celastrol	MS media + 0.3 mg/L IAA	Silver nanoparticles and acetosyringone	Celastrol increased 2.24-fold by treatment with 10 µg mL^−1^ of AgNPs (48 h exposure)	_	3% sucrose pH 5.8 Culture period: 2 weeks	[133]
*Echinacea purpurea* L.	ATCC 43,057	leaf explants	Phenolics Flavonoids caffeic acid derivatives	MS media no PGRs	24-epibrassinolide and l-phenylalanine	1.0 mg/L 24-eBL gave maximum production of phenolics, total flavonoids, cichoric acid, caftaric acid, Echinacoside, and p-coumaric acid.	_	Culture period:21 Days Sucrose: 50 g/L	[115]
*Eurycoma longifolia* J.	*A. rhizogenes* strain A4	Root	9-methoxycanthin-6-one compound	(MS) basal media with no PGRs	MeJA and SA.	0.1 mM MeJA increased production of 9-methoxycanthin-6-one up to three folds as compared to control.	_	pH:4.9 Culture period:12 weeks inoculum size: 0.2 g hairy root in 50 mL of MS basal media	[162]
*Gentiana scabra* B.	*A. rhizogenes* strains ATCC15834	Leaf	Iridoids and secoiridoids	B5 media + 1.0 mg/L NAA, (TDZ), zeatin, IBA	Acetosyringone	Loganic acid increased 6.6- fold in the presence of zeatin (1 mg/L) and gentiopicroside accumulation was 1.8- fold higher in the presence of NAA, 1 mg/L and 1.0 mg/L NAA yield 1.4- and 2.5- fold higher gentiopicroside and swertiamarin.	_	Co-cultivation period: 48 h. Culture period:8 weeks pH:5.7 ± 0.1 N6, WPM, MS and B5 media were tested. B5 liquid media was most suitable	[140]
*Glycyrrhiza glabra* L.	A4	Shoots	glycyrrhizin	^½^ MS medium + 0.1 mg/L IAA	PEG, CdCl2, cellulase, and mannan	1% PEG enhanced glycyrrhizin yield up to 5.4-fold. 200 µg m/L cellulase enhanced glycyrrhizin yield up to 8.6-fold. 10 mg/L mannan enhanced the yield of glycyrrhizin up to 7.8-fold.	_	16 h light/8 h dark period. 100 µM acetosyringone	[163]
*Hybanthus enneaspermus* L.	*A. rhizogenes* strains A4, A4T, 8196 and LBA 9402	Leaf or internode explants	coumarin	MS media+ 0.25 mg/L, 16N-benzyladenine (BA+ 0.1 mg/L (IAA)	Acetosyringone	Coumarin accumulation increased three folds in the superior rhizoclone of A4 origin (A4-HRL-2B7) (3.25 mg/g d.wt. extract) as compared to that in natural roots.	_	Photoperiod: 16 h Culture period: 4 weeks	[164]
*Panax ginseng* C.A.Mey.	*Agrobacterium* *rhizogenes*	Root	ginsenosides	½ MS media + no PGRs	Tween 80	Coumarin accumulation increased three folds in the superior rhizoclone of A4 origin (A4-HRL-2B7) (3.25 mg/g d.wt. extract) as compared to that in natural roots.	_	sucrose: 3% inoculum length: 20 mm long Tween 80: 1.2% *w*/*v* Culture period: 4 weeks	[165]
*Panax quinquefolius* L.	*A.rhizogenes* ATCC 15834	seedlings	ginsenosides	Gamborg media + no PGRs	Yeast extract	(3 days time of exposure and 50 mg/L of YE) increased total ginsenoside content up to 32.25 mg/g D.W	Nutrient sprinkle Bioreactor	Sucrose: 30 g/L YE: 50 mg/L Elicitation period:3 and 7 days Incubation period: 5 weeks	[166]
*Panax vietnamensis* Ha et Grushv.	*Rhizobium rhizogenes* ATCC 15,834 strain	shoot explants	Ocotillol-type ginsenosides	½ MS media + no PGRs	_	With culture conditions the PPD contents evaluated at 0.57% dry weight, the PPT at 0.028% dry weight, and the OCT at 4.3% dry weight in hairy roots.	_	Co-cultivation system sucrose: 3% (*w*/*v*) culture period: 90 days	[167]
*Perovskia abrotanoides* Karel.	ATCC15834 TR105, and R1000	Seedlings and nodes	Cryptotanshinone and tanshinone IIA	MS media No PGR	Acetosyringone	Transformation frequency increased by up to 60.99% when 100 µM of acetosyringone was used. cryptotanshinone and tanshinone IIA levels were 53.17 ± 0.26 and 14.48 ± 0.30 µg/g dry weight, respectively in hairy roots induced by TR105.	_	Half strength MS media Sucrose:3%	[152]
*Plumbago indica* L.	ATCC 15834	Leaf explant	Plumbagin	MS media with no PGRs	Yeast carbohydrate fraction Chitosan Manganese chloride Copper chloride and MeJA	Plumbagin production was enhanced to 1.2–2-fold by treatment with Yeast carbohydrate fraction, chitosan, manganese chloride, copper chloride and MeJA. With 20 days old bioreactor-culture, and exposure of chitosan (200 mg/L) and methyl jasmonate (80 μM) plumbagin production was enhanced to 13.16 ± 1.72 mg g^−l^ dry weight.	Bioreactor with continuous air supply	Bioreactor was maintained in dark at 25 ± 2 °C Sucrose: 3%	[168]
*Polygonum* *multiflorum* Thunb.	*A. rhizogenes* strain KCCM 11879	Leaf	phenolic compounds	MS media + no PGRs	MeJA	Exposure to 50 μM methyl jasmonate for 5 days increased levels of phenolic compounds more than 2.5-fold.	_	Sucrose: 3% Inoculum density: 0.5 g/100 mL Culture period: 21 days	[88]
*Prunella vulgaris* L.,	*A. rhizogenes* (ATCC15834)	Leaf	Rosmarinic acid	MS media + no PGRs	Ethephon and SA	Rosmarinic acid accumulation increased by 1.66-fold 8 days after Eth elicitation and 1.48-fold 2 days post-SA addition.	_	Sucrose: 3% Elicitation period:8 days Culture period: 30 days	[169]
*Stevia* *rebudiana* (Bertoni) Bertoni	A4 strain	Nodal explant	Steviosides	MS media + BAP (0.5–2.0 mg/L) 0.5 mg NAA	Light	Stevioside contents in the SRA4 HR clone on day 75th increased 0.247 ± 0.011 to 1.72 ± 0.052 mg/g dry weight in the root tissues and 0.097 ± 0.072 to 2.12 ± 0.06 mg/L in the media under light conditions	_	co-cultivation period:2–3 days culture period: 75 days agitation rate: 80 rpm ½ MS Media +1.0 mg/LBAP + 0.1 mg/L NAA	[170]
*Talinum paniculatum* Ruiz and Pav	LB510	Leaves	saponin	MS media with no PGRs	-	MS medium supplemented with 5% sucrose and 2.0 strength potassium nitrate of MS, produced the maximum saponin content.	balloon-type bubble bioreactor	Inoculum density: 2 g/400 mL Aeration rate: 0.25 vvm Culture period:14 days	[171]
*Tripterygium wilfordii* Hook. F.,	*A.rhizogenes* ATCC15834	Root	wilforgine and wilforine	MS media + no PGRs	-	10:50 mM NH_4_^+^/NO_3_^−^ and 0.3125 mM phosphate increased wilforgine and wilforine production by 42% and 48%.	_	Incubation period: 7 to 42 days. sucrose: 30 g/L NH_4_^+^/NO_3_: 10:50 mM Phosphate:0.3125 mM pH: 5.8	[158]
*Valerian jatamansi* Jones.	R1601	Young Leaves	Valtrate	MS media No PGR	MeJA JA SA	By treatment with 100 mg/L MeJA, production of Valtrate was increased to a level of 3.63 times, which was higher than non-elicited control.	_	pH: 5.9 Subculturing of hairy roots after every 5 weeks.	[172]
*Withania sominefera* (L.) Dunal	*Agrobacterium tumefaciens* C58C1 (pRiA4)	Leaf	Withaferin A	½ MS media + no PGRs	_	WFA in THRs contain 1.51-fold more WFA (330 ± 0.87 µg/g dry weight (dry weight)) than AHRs (218 ± 0.17 µg/g dry weight)	_	culture period: 40 days biomass doubling time of THRs and AHRs: 18 and 30 days	[173]

## 11. Conclusions

The expeditious advancement in biotechnology has offered adventitious and hairy root cultures as one of the best alternatives to whole plant cultivation for the production of valuable compounds by employing medicinal plants. Plethora of studies has been conducted with the aim to produce valuable compounds by utilizing hairy and adventitious root cultures that have made enormous advances in plant sciences. For maximum production of specific bioactive compounds in these systems, optimization of culture conditions, elicitation strategies, precursor feeding, and various other approaches have been reported in numerous studies. A promising alternative to field cultivation of medicinal plants is adventitious or hairy root culture in a bioreactor system supplied with an elicitor. The commercial exploitation of bioactive substances requires scaling up these approaches utilizing bioreactors. Optimized use of these approaches, either individually or in combination, can provide synergistic effects, resulting in increased biomass productivity and bioactive compounds accumulation. Nevertheless, both chemical and physical optimization is foremost to produce high-quality yields. Furthermore, profound knowledge of biosynthetic pathways of desired compounds in adventitious and hairy roots is still in its early stages. To improve yields metabolic engineering offers an encouraging outlook but requires the understanding of governance on secondary metabolic pathways embroiled on the levels of genes, enzymes, and products including details such as compartmentation and transport is required.

## Data Availability

All the data are included in the present study.
